# *I_h_* Tunes Theta/Gamma Oscillations and Cross-Frequency Coupling In an *In Silico* CA3 Model

**DOI:** 10.1371/journal.pone.0076285

**Published:** 2013-10-18

**Authors:** Samuel A. Neymotin, Markus M. Hilscher, Thiago C. Moulin, Yosef Skolnick, Maciej T. Lazarewicz, William W. Lytton

**Affiliations:** 1 Department of Physiology & Pharmacology, State University of New York Downstate, Brooklyn, New York, United States of America; 2 Department of Neurobiology, Yale University School of Medicine, New Haven, Connecticut, United States of America; 3 Vienna University of Technology, Vienna, Austria; 4 Department of Neuroscience, Uppsala University, Uppsala, Sweden; 5 Medical Biochemistry Institute, Federal University of Rio de Janeiro, Rio de Janeiro, Brazil; 6 Computer Science Program, Brooklyn College, City University of New York, Brooklyn, New York, United States of America; 7 Department of Bioengineering, University of Pennsylvania, Philadelphia, Pennsylvania, United States of America; 8 Department of Neurology, State University of New York Downstate, Brooklyn, New York, United States of America; 9 Kings County Hospital, Brooklyn, New York, United States of America; Georgia State University, United States of America

## Abstract

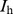
 channels are uniquely positioned to act as neuromodulatory control points for tuning hippocampal theta (4–12 Hz) and gamma (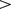
25 Hz) oscillations, oscillations which are thought to have importance for organization of information flow. 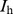 contributes to neuronal membrane resonance and resting membrane potential, and is modulated by second messengers. We investigated 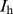 oscillatory control using a multiscale computer model of hippocampal CA3, where each cell class (pyramidal, basket, and oriens-lacunosum moleculare cells), contained type-appropriate isoforms of 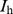. Our model demonstrated that modulation of pyramidal and basket 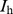 allows tuning theta and gamma oscillation frequency and amplitude. Pyramidal 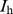 also controlled cross-frequency coupling (CFC) and allowed shifting gamma generation towards particular phases of the theta cycle, effected via 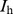 's ability to set pyramidal excitability. Our model predicts that *in vivo* neuromodulatory control of 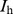 allows flexibly controlling CFC and the timing of gamma discharges at particular theta phases.

## Introduction

The hyperpolarization-activated cyclic-nucleotide gated (HCN) channel is a voltage-gated ion channel involved in sub-threshold resonance [1]–[4]. Additionally, HCN plays an important role in regulating neuronal excitability by setting resting membrane potential (RMP) [5], [6]. HCN produces the current known as 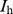 (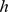 for hyperpolarization-activated), also known as I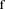 (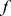 for funny), I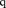 (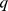 for queer), and as “the anomalous rectifier”. 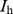 is peculiar/funny/queer/anomalous because, unlike most channels, it inactivates with depolarization (hyperpolarization-activated). Another peculiarity is its mixed permeability, which gives it an intermediate reversal potential (E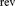) near −30 mV, unlike many channels which are dominated by a major permeability to Na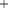, K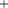, or Ca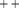.

HCN channels are modulated by cyclic nucleotide second messengers. HCN has four isoforms which are differentially expressed in different cell types and differ in intrinsic properties, kinetics, and pharmacological sensitivities [1], [7]. HCN1 and HCN2 isoforms are the dominant forms in hippocampus, and are present in varying proportions in all cell types studied. Of the two, HCN1 is faster (shorter time-constant).

In addition to its contribution to cell resonance, the HCN channel has a number of properties that suggest 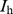 might play a major role in control of oscillations in hippocampus and other brain areas: 1. It is one determinant of a critical cell-excitability control, RMP [5], [8]. 2. It is differentially expressed in different cell types by virtue of inhomogeneous isoform distributions [1], [3], [7], . 3. It is differentially modulated in different cell types by virtue of targeting of particular excitatory or inhibitory cell types by particular neurotransmitters and neuromodulators projecting from different brain areas [2], . Because it is modulated through second messengers, these neurotransmitters and neuromodulators will be expected to have complex interactions within the cell chemistry prior to interacting with the membrane properties via 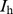 [14].

Hippocampus contains many classes of pyramidal and inhibitory cells, with differing contributions to network dynamics [15], [16]. We hypothesized that differential modulation of 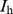 currents in different cell classes would fine-tune the power and frequencies of network-generated oscillations. We therefore investigated the effects of altering 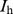 conductance [14], [17] in a computer model of hippocampal CA3, consisting of 800 pyramidal cells, 200 basket interneurons, and 200 oriens-lacunosum moleculare cells [18], using different isoform combinations based on the literature [4], [7], [9]. We found that tuning 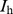 in different cell classes altered network rhythms, providing independent control for gamma and theta oscillations. 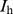 modulation also set the level of cross-frequency coupling and timing of gamma generation relative to the theta cycle. 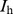 modulation may therefore be an important control point with functional consequences, since these dynamics are hypothesized to contribute to learning and cognitive function [19]–[21].

## Materials and Methods

### Simulations

This model is an extension of a model of hippocampal CA3 that was previously published [18]. Simulations were performed on a Linux system with eight 2.27 GHz quad-core Intel Xeon CPUs using NEURON [22]. Eight seconds of simulation ran in about 2.2 minutes. In order to assess the robustness of the results, we ran each simulation condition with six different randomizations of synaptic inputs, and six different randomizations of network connectivity. Simulations were run in the NEURON simulation environment with python interpreter, multithreaded over 16–32 threads [22], [23]. Analysis of simulation data was done with the Neural Query System [24] and Matlab (Mathworks, Inc.). The full model is available on ModelDB (https://senselab.med.yale.edu/modeldb).

### Cells and connections

**Figure 1:**
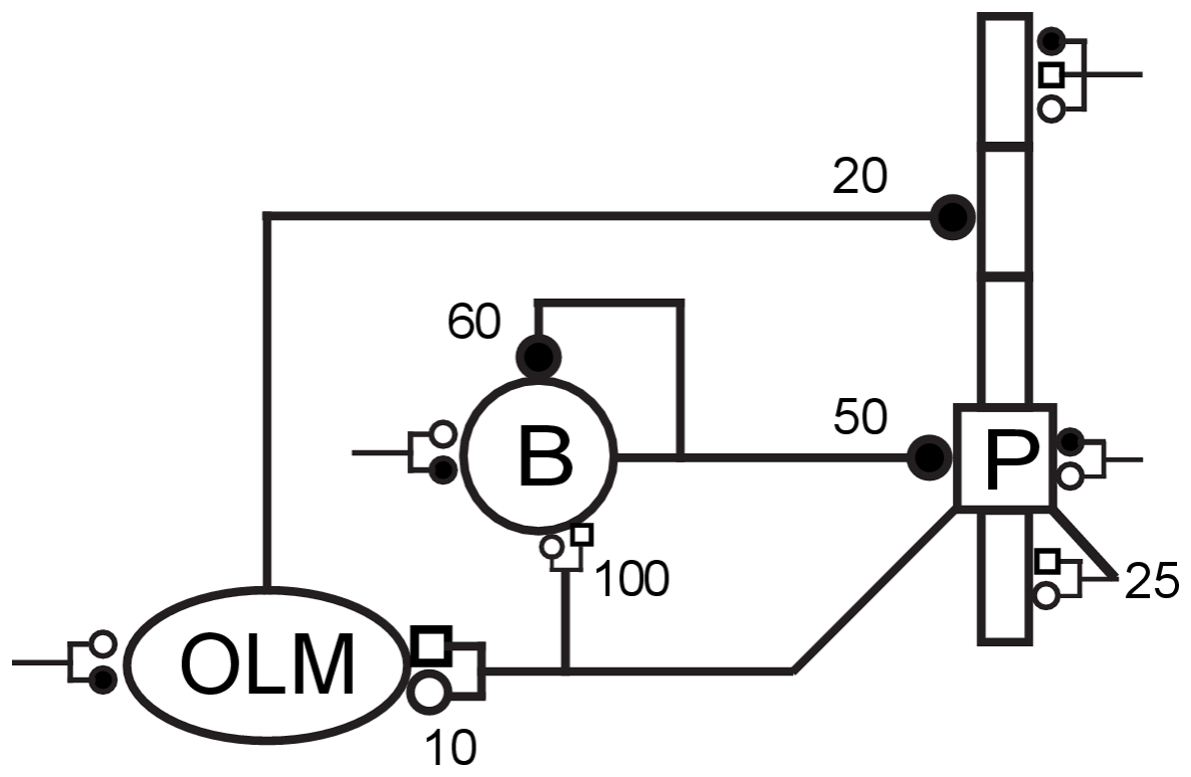
Schematic representation of the network. Each symbol represents a population: 800 pyramidal cells (P), 200 basket cells (B), 200 OLM cells. Convergence values (number of inputs for an individual synapse) are shown near synapses: GABA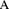 receptors (filled circles), AMPA receptors (open circles), NMDA receptors (open squares). External stimulation from other areas was modeled by synaptic bombardment (synapses with truncated lines).

The network consisted of 800 five-compartment pyramidal (PYR) cells, 200 one-compartment basket (BAS) interneurons, and 200 one-compartment oriens lacunosum-moleculare (OLM) interneurons [25]–[27] (Fig. 1). Current injections (pyramidal cell s: 50 pA; OLM cells −25 pA) were added to get baseline activity. This was a simplification to substitute for absence of external inputs from other areas, and to compensate for the small size of the model, which did not allow for much self-activation.

All cells contained leak current, transient sodium current 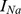, and delayed rectifier current 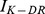, to allow for action potential generation. Additionally, pyramidal cells contained in all compartments potassium type A current 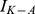 for rapid inactivation, and hyperpolarization-activated current 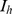 based on HCN2 isoform parameterization [3], [7]. Interneurons contained hyperpolarization-activated 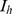 current based on HCN1 isoform parameterization [3], [7], [9]. The OLM cells had a simple calcium-activated potassium current 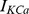 to allow long lasting inactivation after bursting, high-threshold calcium current 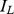 to activate 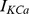, hyperpolarization-activated current 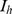, and intracellular calcium concentration dynamics. Selection of currents was based on prior published models [25], [28]–[30] and basket interneuron 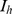 currents were based on the literature [3], [7], [9].

For all cell types the 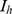 current was defined as 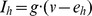, where 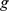 is the instantaneous conductance, 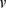 is the membrane potential, and 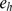 is the reversal potential (−30 mV for BAS and PYR cells; −40 mV for OLM cells). Each 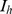 channel had a parameter, 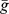, which represented the maximal conductance density (0.0002 S/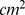 for BAS, 0.0001 S/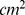 for PYR, and 0.00015 S/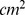 for OLM cells). To simulate neuromodulatory scaling of the 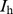 conductance values, 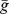 was multiplied by another factor, 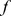, which varied between 0.0 and 2.0, and was set to 1.0 for the baseline simulations. Instantaneous conductance was then set to 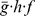, where 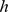 is the 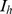 gating variable which activated at hyperpolarized voltages. The evolution of the 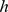 state variable in time followed 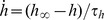, where 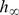 was the voltage-dependent steady-state value of 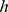, and 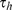 was the voltage-dependent time-constant of 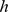 (in milliseconds).

For BAS cells, 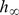 was set to 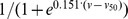, where 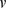 was the membrance voltage, and 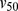, the 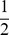-maximal voltage level, was set to −73 mV. BAS cell 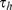 followed 
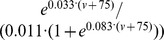
. PYR 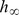 followed 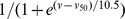, with 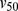 at −82 mV. PYR 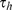 was set to 
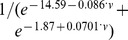
. OLM 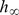 followed 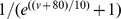, and OLM 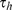 followed 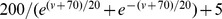.

#### 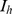-static

To test the effect that 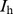 had on individual neurons, we isolated the dynamic component, which had the voltage-dependent conductance (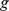) described above. To do this, we first ran a set of 7 second simulations, varying the 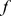 parameter from 0.0 to 2.0 (with increments of 0.5) and measured the 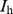 conductance (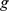) at the end of each simulation. This conductance (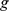) was saved for each compartment of each cell type. 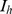-static was then defined as the current from a leak channel with conductance equal to 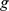 measured in the previous step, and with the same reversal potential (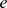) as the original 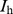 channel. 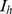-static followed 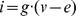.

The network contained 152,000 synapses. Pyramidal cell projections were mixed alpha-amino-3-hydroxy-5-methyl-4-isoxazolepropionic acid (AMPA) and N-methyl-D-aspartic acid (NMDA) response. Basket cells synapsed on the soma of both pyramidal cells and other basket cells via gamma-aminobutyric acid A (GABA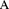) receptors. OLM cells connected to distal dendrites of pyramidal cells via GABA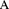 receptors. AMPA and NMDA receptors had reversal potentials of 0 mV, while GABA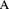 receptors had reversal potentials of −80 mV.

Connections in the network were set up based on fixed convergences (Table 1). However, connectivity was random and specific divergence could therefore vary. All synaptic delays between cells were 2 ms, to simulate axonal propagation and neurotransmitter diffusion and binding, which were not explicitly modeled. Parameters were based on the literature where available, as well as on previous models [25], [31].

**Table 1:** Synaptic parameters.

Presy naptic	Postsy naptic	Receptor	τ_1_ (ms)	τ_2_ (ms)	Conductance (nS)	Conver gence
Pyramidal	Pyramidal	AMPA	0.05	5.3	0.02	25
Pyramidal	Pyramidal	NMDA	15	150	0.004	25
Pyramidal	Basket	AMPA	0.05	5.3	0.36	100
Pyramidal	Basket	NMDA	15	150	1.38	100
Pyramidal	OLM	AMPA	0.05	5.3	0.36	10
Pyramidal	OLM	NMDA	15	150	0.7	10
Basket	Pyramidal	GABA_A_	0.07	9.1	0.72	50
Basket	Basket	GABA_A_	0.07	9.1	4.5	60
OLM	Pyramidal	GABA_A_	0.2	20	72	20

### Synapses

Synapses were modeled by a standard NEURON double-exponential mechanism with parameters based on Tort *et al.*, 2007 [25] (Table 1). Magnesium block in NMDA receptors used the experimental scaling factor 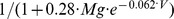; 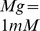 [32].

### Background activity

Throughout the simulation duration, background activity was simulated by synaptic excitatory and inhibitory inputs following a Poisson process, sent to somata of all cells and dendrites of pyramidal cell s (Table 2). Fast background activity consisted of AMPA and GABA-ergic bombardment at 1000 Hz. Slow activity used activation of the NMDA receptors at a mean frequency of 10 Hz. These inputs represented the influence of surrounding excitatory and inhibitory cells not explicitly modeled in the simulation and produced a high conductance state similar to that observed *in vivo* [33]. In addition, we placed slow excitatory inputs in the last distal apical compartment of pyramidal cells, in order to model input from the entorhinal cortex. This input was capable of simulating calcium-spike-like activity in the dendritic compartment and driving sparse firing of pyramidal cells. Synapses were activated randomly according to a Poisson distribution.

**Table 2:** Parameters for modeling background activity.

Cell	Section	Synapse	τ_1_ (ms)	τ_2_ (ms)	Conductance (nS)
Pyramidal	Soma	AMPA	0.05	5.3	0.05
Pyramidal	Soma	GABA_A_	0.07	9.1	0.012
Pyramidal	Dend	AMPA	0.05	5.3	0.05
Pyramidal	Dend	NMDA	15	150	6.5
Pyramidal	Dend	GABA_A_	0.07	9.1	0.012
Basket	Soma	AMPA	0.05	5.3	0.02
Basket	Soma	GABA_A_	0.07	9.1	0.2
OLM	Soma	AMPA	0.05	5.3	0.0625
OLM	Soma	GABA_A_	0.07	9.1	0.2

Local field potential (LFP) was simulated by a sum of differences in membrane potential between the most distal apical and the basal dendritic compartment over all pyramidal cells. Before calculating spectral power, the DC component of the signal was removed [34]. In addition, the first and last 200 ms of simulated data were removed to avoid artifacts associated with endpoints in the data. The spectral power was calculated using the multitaper method (MatLab pmtm() function; Mathworks, Inc.). Peak values in the power spectra are reported for theta (4
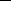
12 Hz) and low gamma (25
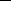
55 Hz) frequency bands. All 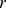-values reported were calculated using the Pearson correlation coefficient. To determine cross-frequency-coupling (CFC) between theta and gamma oscillations, we used a modified version of the modulation index [35] to reduce artifacts in CFC measures associated with sharp spikes [36]. Theta oscillations were extracted by filtering LFPs between 6–10 Hz using a zero phase distortion band-pass filter. Gamma spikes (duty cycle between 18–40 ms, corresponding to 55–25 Hz) were extracted using a time-domain feature-extraction method [37]. Theta phases at times of gamma spike peaks were then used to form the gamma-amplitude/theta-phase measure, which consisted of 100 equally-spaced phase bins, and were then used to calculate the modulation index [35].

Final evaluations to produce the results presented here were made over the course of 1044 network simulations, using six different random wirings, six different input streams, and variations in maximal 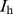 conductance level (relative to baseline: 0.0, 0.5, 1.0, 1.5, 2.0) at the different cell types, where baseline is the 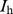 density estimated from the literature. A typical network simulation (8 s; 1200 neurons) took approximately 2.2 minutes using 16 threads on a 2.27 GHz Intel Xeon quad core CPU.

A long-duration simulation set (900 seconds for each simulation) was run using 5 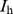 levels for the pyramidal and basket cells. These simulations all had identical wiring and input streams. The data obtained were used to evaluate theta/gamma cross-frequency-coupling and phase relationships as a function of 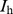 level.

An additional set of simulations of isolated cells was run, varying 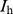 conductance level in the same amounts as in the network. These simulations were used to assess 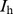 effects on resting membrane potential (RMP) and synaptic integration. These simulations were run for 7 s to allow the cells to reach a steady-state where net transmembrane currents were zero. Then, 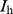 conductance was measured and was used to set a fixed conductance with equivalent E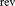 to 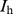, to separate 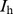 dynamics from its static features. In these simulations, AMPA and GABA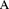 inputs (0.5 nS) were provided at 5.5 s to assess post-synaptic-potential amplitude and temporal integration.

## Results

This study involved over 1000 eight-second network simulations, testing six different input streams, and variations in maximal 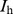 conductance level for the different cell types. These are presented as 0.0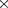 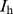, 0.5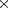 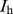, 1.0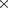 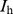, 1.5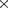 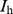, 2.0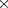 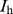, relative to a baseline set to a standard 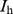 density estimated from the literature. In order to ensure robustness of the results shown, each simulation was tested with six different wirings (wiring density is parameterized but specific point-to-point wiring is random). An additional set of 25 long-term (900 second) simulations were run to evaluate theta/gamma cross-frequency-coupling and phase relationships as a function of 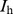 level. Simulations were run using the NEURON simulator on Linux on a 2.27 GHz quad-core Intel XEON CPU. Eight seconds of network simulation ran in 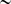
2.2 minutes.


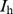
 is a prominent part of resting conductance, contributing to resting membrane potential (RMP), due to the presence of non-zero 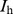 conductance at RMP, and to a relatively depolarized reversal potential (E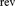). The isolated model oriens-lacunosum moleculare (OLM) cell was depolarized with increasing 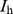, from −68.1 mV without 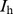, to −64.3 mV at 0.5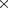, to −61.8 mV at 1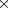 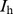. Increasing 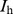 past baseline produced further depolarization and cell firing. At 1.5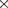 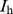, the OLM produced a single action potential and then stabilized with an RMP of −59.5 mV. Further increase to 2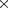 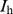 produced rhythmic firing at 6 Hz, a low theta frequency. Pyramidal (PYR) and basket (BAS) cells displayed monotonic RMP dependence on 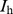, with RMP ranging from −65.6 – **–**57.5 mV and −65 – −61.7 mV, respectively. PYR cells emitted one and two transient spikes at 1.5 and 2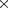 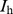, respectively, while BAS cells did not exhibit any spontaneous firing.

Altering 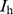 altered both the magnitude and time-course of excitatory and inhibitory postsynaptic potentials (EPSPs and IPSPs). Both types of PSP showed increasing amplitude with increasing 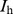. IPSP amplitude increase can be directly explained as a consequence of the greater driving force at the more depolarized RMP. With 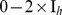, BAS and PYR increases were from 0.30–0.44 mV and 0.49–1.1 mV, respectively, while OLM increased from 0.22–0.65 mV, with 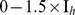 (at 2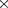 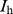, OLM fired rhythmically, precluding accurate IPSP measurement).

**Figure 2:**
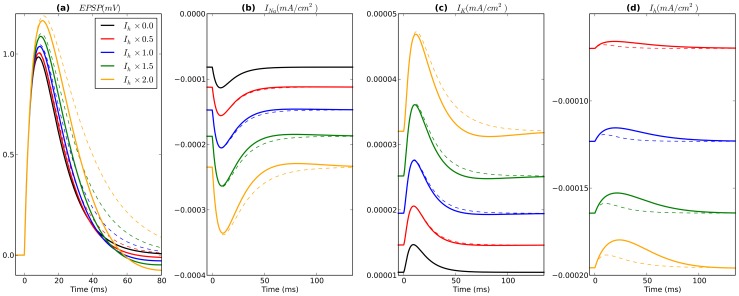
BAS cell response to AMPA stimulus at different levels of 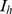 conductance. Solid lines represent responses with dynamic 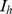 and dotted lines represent responses with static 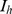. Note that only BAS cell is displayed since it did not fire action potentials in response to AMPA-ergic stimulation. Time axes are relative to AMPA input at 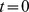 ms. (**a**) EPSP (starting voltage levels aligned vertically for easier comparison of EPSPs), (**b**) 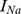, (**c**) 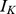, and (**d**) 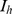 at BAS cell soma.

EPSP amplitude was also generally augmented with 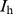 increase (Fig. 2a). This is a paradoxical effect, given that the direct RMP depolarizing shift that augmented IPSP driving force decreased EPSP driving force. In addition to reducing driving force, increased 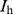 also increased shunting, an effect that would reduce amplitude of both EPSPs and IPSPs. Both of these static factors predict EPSP amplitude *decrease*. We therefore predicted that replacement of the dynamical 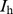 with a static version (fixed conductances of equivalent magnitudes and E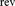; see Materials and Methods) would reduce EPSP amplitude. Instead, we found even larger increases in EPSP magnitude. Examination of transmembrane current activations of both Na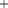 and K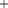 currents, revealed a larger depolarizing effect of 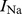 (Fig. 2b), which dominated over the hyperpolarizing effect of 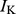 (Fig. 2c). With block of Na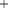 and K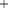 channels, EPSP amplitudes decreased with depolarized RMP, as originally predicted. The dynamics of 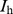 itself worked to reduce this amplitude increase: 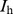 turns off during the EPSP, reducing the degree of depolarization and reducing the 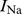 boost (Fig. 2d). The combination of these 5 effects (driving force,shunting, 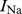, 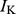, 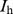 dynamics) produced a mild overall EPSP amplitude increase, that was far less pronounced than the increase in IPSP: BAS: 0.99–1.17 mV with 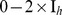; PYR 1.78–2.21 mV with 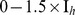, 2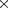 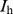 produced spiking; OLM 0.96–1.07 mV, with 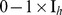, 1.5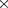 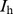 produced spiking. In one case, a slight decrease in EPSP amplitude was seen: 1.78 to 1.72 mV with increase of 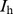 from 0 to 0.5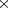 baseline in the PYR cell.

Time to peak PSP was delayed by increasing 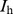. These effects were again a result of multiple conflicting tendencies. We therefore looked separately at the effects of the conductance change, effects of other channels, and effects of 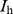 dynamics themselves. The conductance change alone lowered R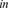 which reduced membrane time-constant, which reduced the duration of synaptic response, leading to an earlier peak. Returning Na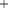 and K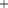 currents to the simulation moved PSP peaks to slightly later times. Adding back the dynamics of 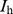 moved the PSPs to earlier times again. With all these dynamical factors in place, IPSP delays had noticeably increasing values: BAS 10.8–12.5 ms with 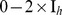; PYR 6.8–9.5 ms with 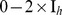; OLM 10.0–16.5 ms with 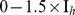 since 2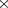 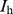 produced rhythmic spiking. Similar effects were observed for EPSP delays (BAS: 8.6–10.7 ms with 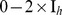; PYR: 4.9–8.1 ms with 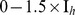 since at 2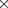 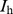 the synaptic input produced a spike; OLM: 7.6–9.5 ms with 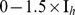).

**Figure 3:**
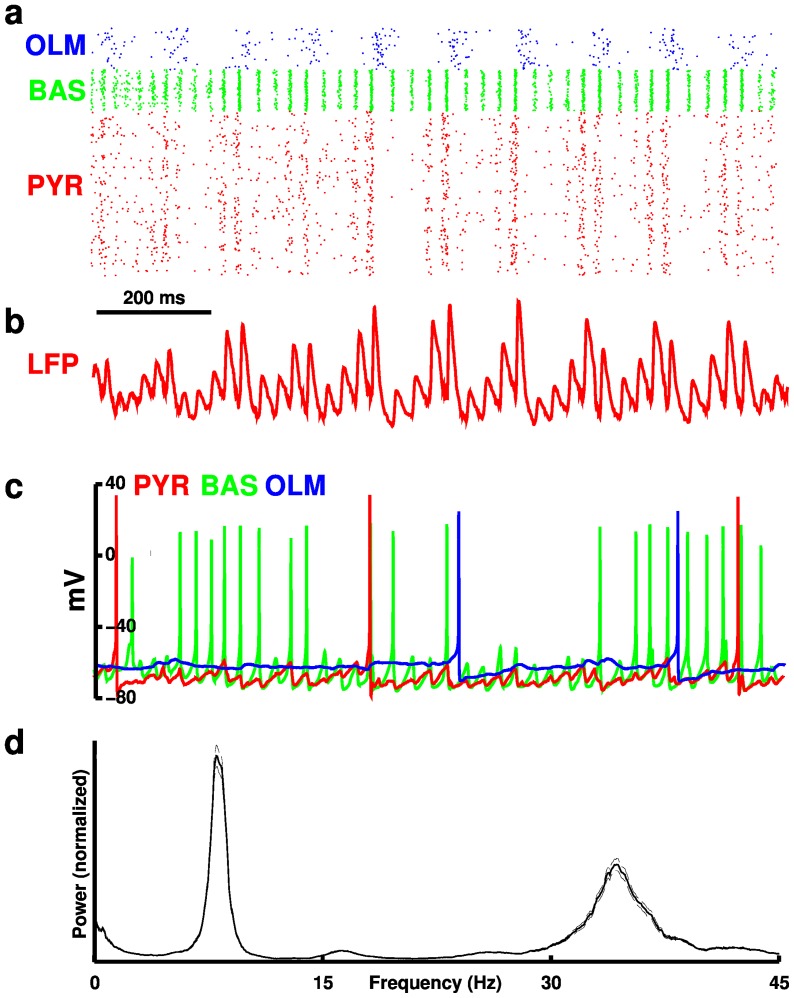
Activity of network at baseline. (**a**) Raster plot showing firing times of cells within the network. Cell types are color-coded. (**b**) Local field potential (LFP) generated by PYR cells. (**c**) Voltage traces from soma of different cell types. (**d**) Average (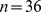) local field potential power spectrum 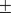 standard error of the mean (SEM; dotted lines).

In the network, baseline firing rates of PYR, BAS, and OLM cells were 1.8 Hz, 10.8 Hz, and 1.2 Hz, respectively. As a population, OLM cells tended to fire rhythmically at theta frequency (4
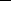
12 Hz). Interactions between cells in the network led to the generation of theta and gamma (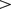
25 Hz) oscillations (Fig. 3). These emergent rhythms were generated through the different synaptic time constants in the network and through the cellular interactions of pyramidal-interneuron network gamma (PING) and interneuron network gamma (ING) [15], [31], [38], [39].

Baseline oscillations were similar to those described in earlier versions of this model, which contained 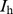 currents in PYR but not in BAS cells [18]. Briefly, strong periodic OLM firing shut down PYR activity resulting in lower PYR 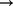 BAS drive. PING interactions between PYR and BAS cells contributed to gamma oscillations: lower PYR to BAS drive led to lower gamma amplitude during periods of OLM 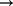 PYR inhibition. As the PYR cells recovered from OLM inhibition, their activity gradually built up providing increased drive to BAS cells and increasing gamma amplitudes, accounting for nesting of gamma within the theta cycle (Fig. 3a,b). ING also contributed to the strength of gamma in this model due to strong BAS 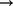 BAS connectivity. The presence of 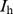 led to a slightly higher gamma amplitude than in the prior model due to the stronger repolarization enhancing the ING mechanism. Individual cell voltages showed multiple rhythms as well, with both the PYR and BAS cells reflecting the network oscillation in their postsynaptic potentials (Fig. 3c).

**Figure 4:**
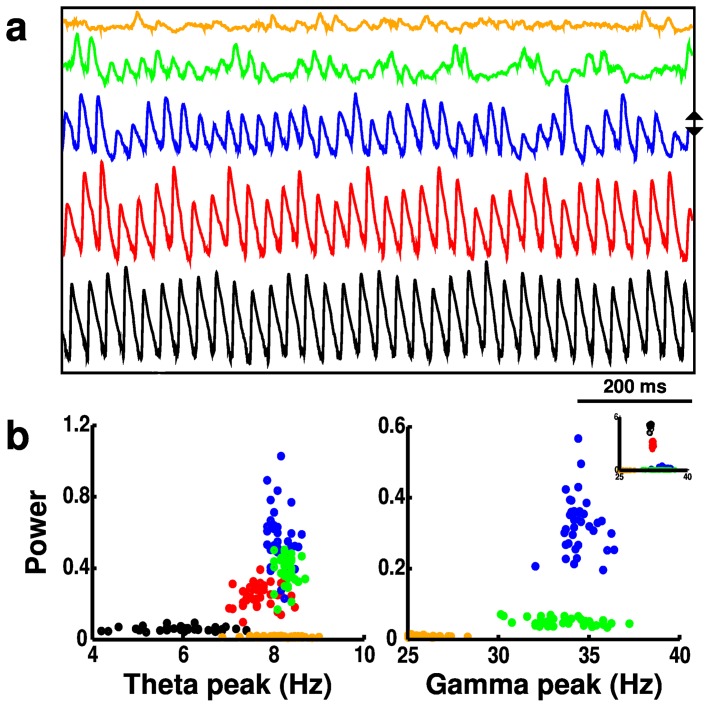
Activity (from 180 simulations) with 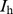 scaling in OLM interneurons. (**a**) Local field potentials (LFPs). Blue LFP is from baseline simulation. Up (down) arrows indicate directions of increase (decrease) of 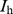. (**b**) Scatter plots of theta (left) and gamma (right) peak frequencies and power (normalized); color code as in (**a**); each point from a single simulation with different random activation and wiring. Gamma: main panel shows zoom-in of subset of values. Inset shows full set.

**Figure 5:**
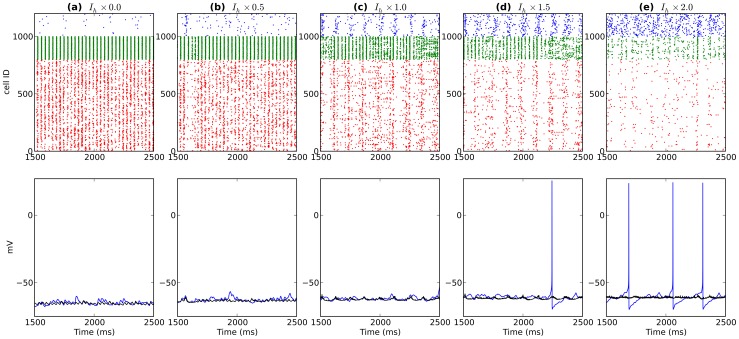
Activity from a single network after modulating OLM 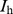 levels (OLM 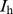 increases left to right). Top shows spike rasters (PYR:red; BAS:green; OLM:blue). Bottom displays somatic voltage from a single OLM cell (blue) and average somatic voltage from 200 OLM cells (black).

Given the complex of RMP shifts and temporal integration properties through PSP alterations in the individual cells, we hypothesized that 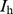 changes would substantially alter frequency and power in network rhythms. Testing 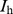 modulation across different cell types within the full network demonstrated consistent but dramatically different effects depending on which cell type was targeted. We started by looking at OLM cells because they provide a central modulating role for theta activity (Fig. 4, Fig. 5) [18]. Reducing or eliminating 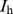 from OLM cells abolished theta by eliminating the depolarizing influence of 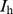. The resulting hyperpolarization reduced OLM firing rate (1.2
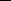
0.2 Hz) which reduced theta modulation throughout the network (red and black in Fig. 4a,b; Fig. 5a,b). The reduced inhibition coming from OLM cells resulted in higher firing rates of PYR cells (1.8
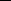
3.5 Hz), which then strengthened BAS activity (10.8
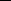
28.7 Hz). The increased dominance of PYR and BAS populations produced a large increase in gamma power (inset in Fig. 4b right) created via the PING mechanism. Increasing OLM 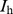 conductance from baseline increased OLM firing rate (1.2
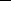
2.8 Hz) and caused the OLM inhibition of the network to dominate, gradually reducing both theta and gamma power as PYR and BAS rates went towards zero (PYR:1.8
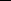
0.4 Hz; BAS:10.8
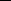
2.0 Hz; Fig. 5e).

Increasing 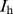 conductance across all cellular locations produced effects primarily similar to the effects on OLM, with reduced theta power and augmented gamma at reduced 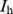 amplitudes. These effects were brought about via the strong governing inhibitory influence of OLM cells, which increased at heightened 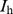 levels. As with OLM 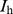 enhancement, higher 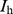 values showed decrease in gamma power and frequency with increase in theta.

**Figure 6:**
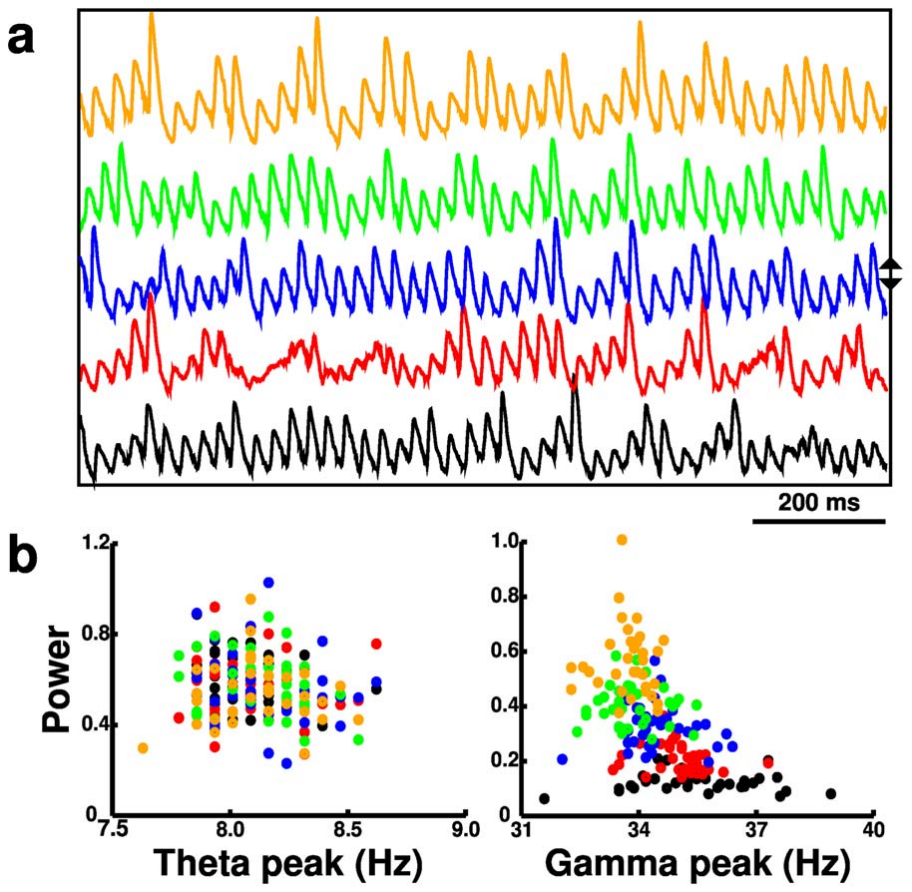
Activity (from 180 simulations) with 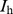 scaling in basket (BAS) interneurons. (**a**) Local field potentials (LFPs). Blue LFP is from baseline simulation. Up (down) arrows indicate directions of increase (decrease) of 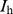. (**b**) Scatter plots of theta and gamma peak frequencies and power (normalized).

**Figure 7:**
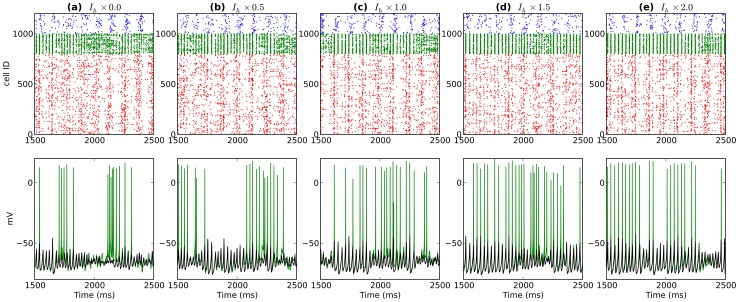
Activity from a single network after modulating BAS 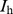 levels (BAS 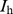 increases left to right). Top shows spike rasters (PYR:red; BAS:green; OLM:blue). Bottom displays somatic voltage from a single BAS cell (green) and average somatic voltage from 200 BAS cells (black).

BAS cells are particularly involved in both ING (BAS-BAS) and PING (PYR-BAS) mechanisms of gamma generation [38], [39]. Hence, it was not surprising that variation of BAS 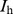 altered gamma power and frequency consistently with no consistent effect on theta (Fig. 6, Fig. 7). Increased BAS 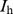 augmented gamma power (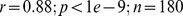) and reduced gamma frequency (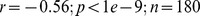). The increased power corresponded to increase in the BAS population firing rates (9.1
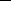
12.6 Hz with 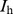 0
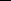
2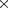) due to the depolarizing effect of 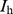. These increases in BAS firing also dampened PYR firing (1.8
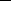
1.7 Hz), which secondarily reduced OLM activity (1.3
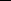
1.2 Hz). The decreased gamma frequency was due to the longer synaptic integration times that the BAS cells displayed with enhanced 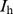.

**Figure 8:**
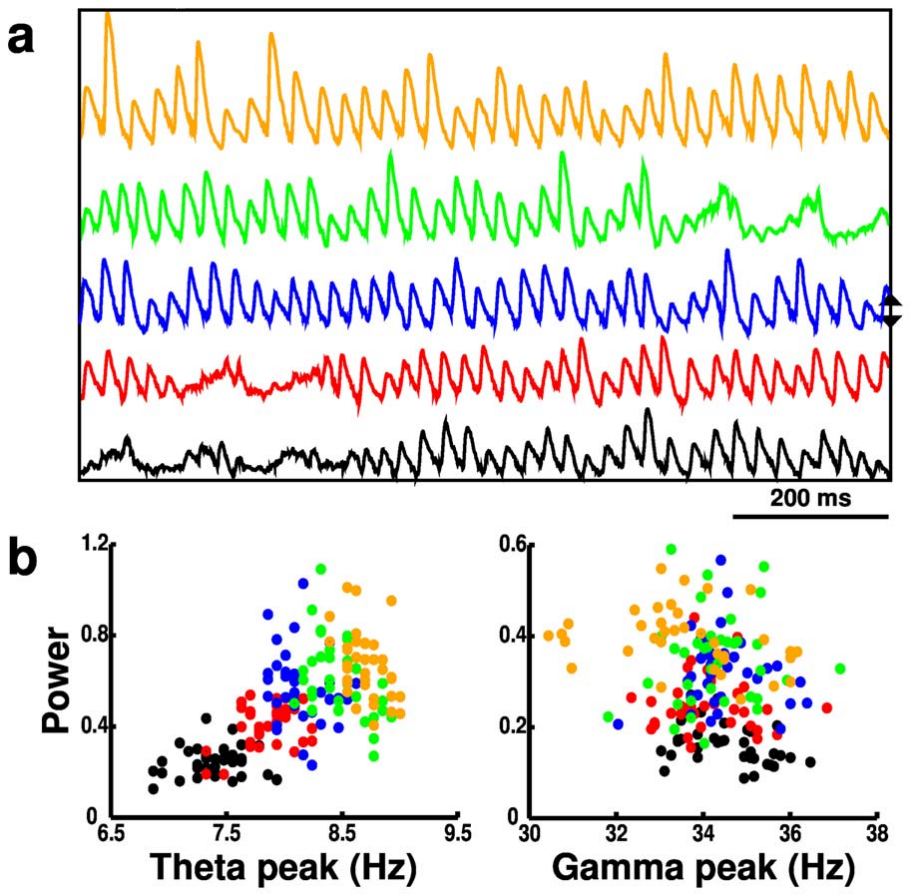
Activity (from 180 simulations) with 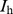 scaling in pyramidal (PYR) cells. (**a**) Local field potentials (LFPs). Blue LFP is from baseline simulation. Up (down) arrows indicate directions of increase (decrease) of 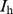. (**b**) Scatter plots of theta and gamma peak frequencies and power (normalized).

**Figure 9:**
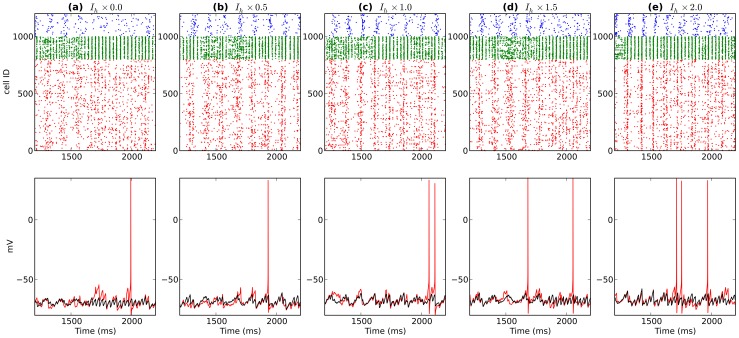
Activity from a single network after modulating PYR 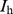 levels (PYR 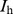 increases left to right). Top shows spike rasters (PYR:red; BAS:green; OLM:blue). Bottom displays somatic voltage from a single PYR cell (red) and average somatic voltage from 800 PYR cells (black).

By contrast with BAS 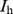 effects, PYR 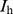 effect was primarily on theta, progressively increasing theta peak (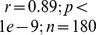) and power (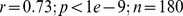; Fig. 8, Fig. 9; consistent with experiment [40]). Increases in theta peak and power were effected through increased PYR firing (1.6
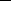
1.9 Hz) which produced increased OLM firing (0.9
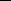
1.6 Hz). Unlike in Fig. 4, OLM firing did not suppress PYR firing since PYR activity was the driving force and was supported by the PYR 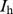. Due to PING interplay, gamma oscillation power was positively correlated with PYR 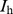 level (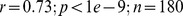; BAS rates: 9.2
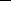
12.1 Hz). Although gamma peak frequency was not significantly shifted, there was some broadening with increasing PYR 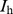. Overall PYR 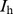 modulation tuned both theta and gamma power together, distinct from other pharmacological effects where theta and gamma are inversely correlated [18].

**Figure 10:**
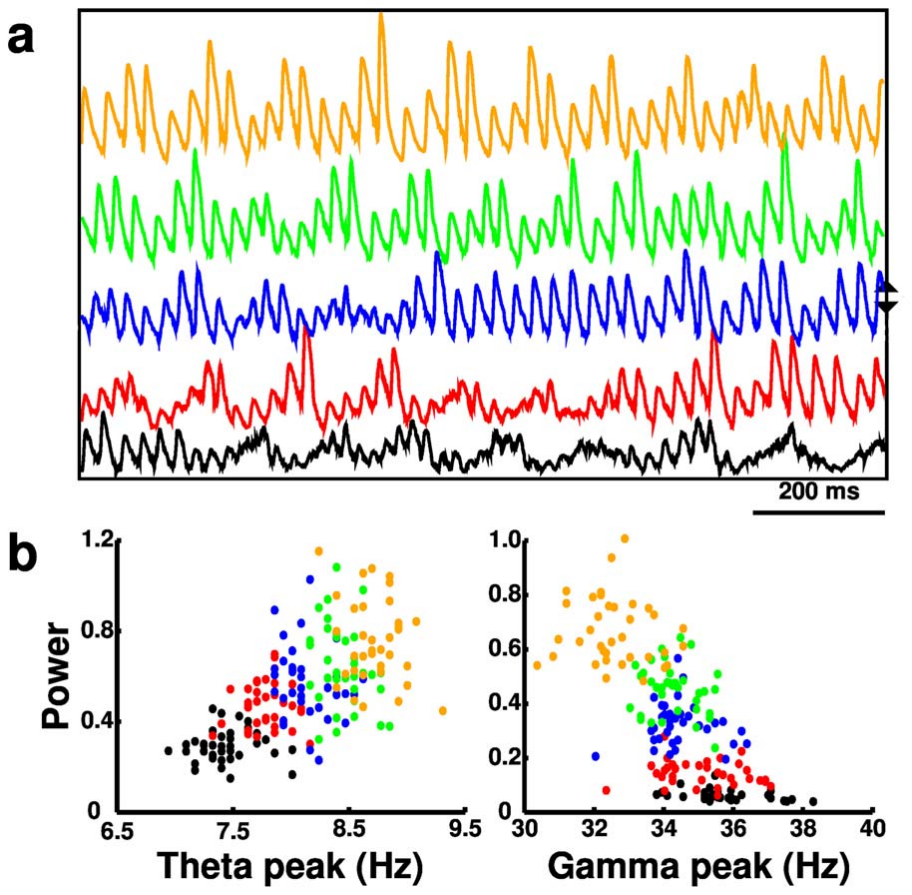
Activity (from 180 simulations) with 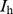 scaling in both pyramidal (PYR) and basket (BAS) cells. (**a**) Local field potentials (LFPs). Blue LFP is from baseline simulation. Up (down) arrows indicate directions of increase (decrease) of 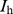. (**b**) Scatter plots of theta and gamma peak frequencies and power (normalized).

**Figure 11:**
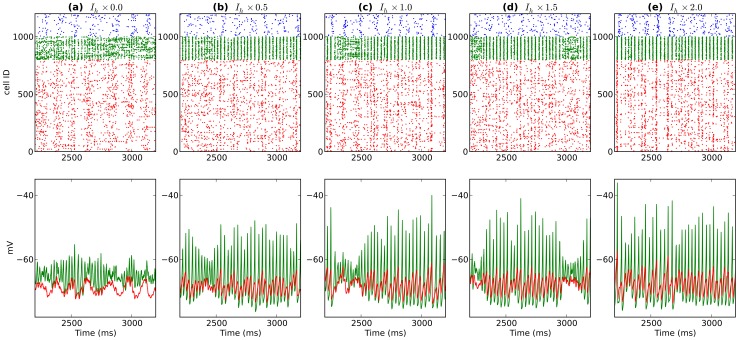
Activity from a single network after modulating PYR and BAS 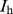 levels (PYR and BAS 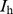 increases left to right). Top shows spike rasters (PYR:red; BAS:green; OLM:blue). Bottom displays average somatic voltage from PYR (red; 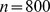) and BAS (green; 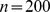) cells.

The contrast of a nearly orthogonal arrangement of strong influence of PYR 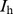 on theta and strong influence of BAS 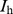 on gamma led us to hypothesize that detailed control of network oscillation could be effected through comodulation of 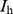 in both. This comodulation could involve simultaneous control where 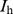 in both cell types were altered together. Alternatively, more complex modulation could occur via activation through different second messengers, or different isoform second-messenger sensitivity, through activation by a neuromodulator with divergent downstream effects. Simultaneous 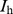 modulation of both PYR and BAS cells produced an additive effect, with changes in both theta and gamma rhythms (Fig. 10, Fig. 11). There was a clear trend of progressively increasing theta peak (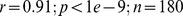) and a similar trend for increasing theta power (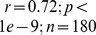). The changes in theta power were brought about by increased PYR firing (1.6
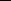
1.9 Hz) which drove increases in OLM firing (0.9
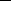
1.6 Hz). Similar to the simulations where PYR 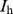 was modulated independently, OLM firing did not suppress PYR firing due to 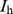 increases supporting PYR activity. Gamma oscillation power had a large positive correlation with PYR and BAS 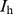 levels (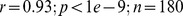) due to direct enhancement to BAS population activity via 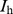 (8.0
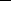
14.1 Hz) and also secondarily due to PING mechanisms. Gamma peak frequency had a clear trend of reduction with increases in PYR and BAS 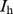 (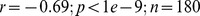), due to the extended delays to peak IPSPs and EPSPs that PYR and BAS cells exhibited with increasing 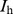.

**Figure 12:**
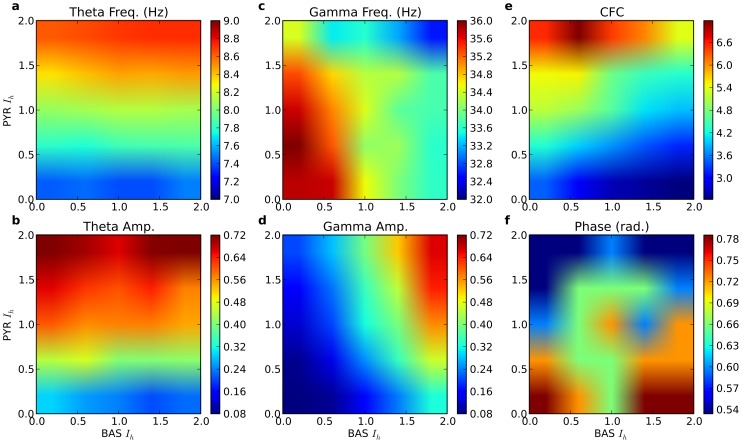
Amplitudes and coupling of oscillations with variation of 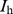 density in BAS and PYR cells (x- and y-axes, respectively). (**a**) Theta frequency and (**b**) amplitude are controlled by PYR 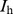, while (**c**) Gamma frequency and (**d**) amplitude are largely controlled by BAS 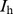. (**e**) Cross-frequency coupling (gamma amplitude modulation by theta phase) is greatest when theta is strong (high PYR 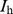) with gamma relatively weak. Units are scaled up by 1e3 for readability. (**f**) Gamma amplitude peaks in the region between 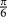 (0.5) and 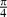 (0.8) radians in a complex pattern. (a,b,c,d: average of 900 8s simulations; e,f: average of 25 900 s simulations).

HCN1 and HCN2 have different molecular modulators: cAMP selectively modulates HCN2, [41], [42] while p38 MAP kinase modulates HCN1 [43]. However, the complexity of linkages from neuromodulators to expression of second and third messengers, and the consequent control in HCN isoforms by these messengers, is currently inaccessible to simulation. We therefore assessed all combinations of 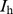 modulation at PYR and BAS cells in order to observe the patterns of gamma-theta relations that could be expressed through HCN modulation in this system. As expected from the relative independence of gamma and theta control from the cell types, we found that these patterns were highly constrained (Fig. 12). Both theta amplitude and frequency increased with PYR 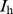 level with effectively no effect of BAS 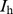 levels (Fig. 12a,b).

Although gamma frequency (Fig. 12c) and amplitude (Fig. 12d) showed primary control by BAS 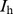 as expected, there was also a prominent effect of PYR 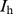, producing the greatest overall gamma amplitude augmentation with coordinated increase in both BAS and PYR 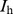. Hence the highest gamma amplitude and highest gamma frequency also showed correlation with the highest theta amplitude and frequency.

Cross-frequency-coupling (CFC) measures the ability of the slower theta wave to provide an envelope that modulates the amplitude of the superimposed faster gamma. Since the strong OLM inhibition only allowed co-expression of theta and gamma oscillations in a relatively narrow range of OLM 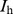, we only measured CFC as a function of PYR and BAS 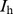. Substantial CFC was only present with high PYR 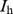, corresponding to large theta (Fig. 12e). The difference between low and high CFC can be seen in Fig. 10a. The black trace demonstrates low CFC: at left only a little alteration of gamma amplitude with theta is seen; at right there is almost no gamma hence no coupling. By contrast the orange trace shows substantial coupling, most readily seen in the 4th theta cycle. Note that these cycle-to-cycle differences make the overall CFC difficult to calculate. In this high PYR 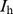 regime, coupling was highest at low values of BAS 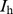, where average gamma activity, reflecting this modulation from low to high, was low (Fig. 12d). By contrast high BAS 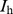 corresponded to a strong continuous gamma which was not as readily modulated. Peak coupling corresponded to oscillations with gamma frequency of 33.5 Hz and theta frequency of 8.6 Hz.

Across 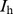 levels, the peak gamma amplitude always occurred during the positive portion of the theta cycle (Fig. 12f), slightly after the theta peak from 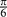 to 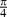 radians (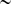
0.5–0.8, where 0 is theta peak). This is consistent with experimental data, which shows peak amplitude of gamma occurring on the positive but descending portion of the theta oscillation [44]. Increased PYR 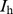 shifted peak gamma amplitude towards earlier phases of the theta cycle. This was due to the depolarizing effects of PYR 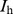 producing heightened PYR excitability, leading to earlier PYR cell firing, and hence earlier production of gamma via PING. Reduced phase lag was therefore associated with stronger CFC (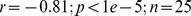).

At baseline, PYR spiking tended to occur near the peak of theta (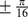 radians), earlier than the theta phase for maximum gamma. This delay from peak PYR firing to peak local field gamma is consistent with a PING mechanism: peak PYR firing engages a larger number of inhibitory cells. This then leads to a subsequent peak gamma cycle, representing the maximum proximal/distal synaptic-activation differences, which then occurs on the subsequent cycle.

## Discussion

Our modeling predicts that neuromodulation of 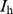 conductance could have several functional roles in *in vivo* neuronal dynamics including: 1) tuning of theta and gamma oscillation amplitude and frequency, 2) modulation of cross-frequency coupling (CFC) levels, and 3) enhanced excitability of cells within a circuit, expressed as increased gamma oscillation amplitude at earlier phases of the theta cycle. 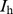 is uniquely positioned for these roles for several reasons: 1) 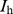 enhances resonance in individual neurons, 2) 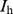 contributes to resting membrane potential, and hence neuronal excitability, 3) multiple HCN isoforms are differentially expressed in different cell types known to contribute to different oscillation frequencies, and 4) neuromodulators allow precise control of the conductance of specific HCN isoforms via second-messenger signaling cascades [7], [43]. These functions of theta and gamma oscillations are linked to different aspects of cognition and behavior: CFC level is correlated with hippocampal-dependent learning performance [21], [45] and attentional modulation [46], and gamma nesting within theta oscillations is a hypothesized mechanism for encoding information dynamically [20].

We investigated 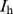 channel function in a multiscale model across levels from ion channel population to the neuronal network. Emergent predictions arose at the levels of channel interactions in dendrites, of dendritic signal interactions in cells and of neurons forming the network. At the dendritic and cellular level, 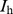 generally increased both EPSP and IPSP magnitude and duration with some variation by cell type. At the cell level, excitability increased due to cell depolarization. At the network level, 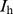 modulation altered both theta and gamma, with effects depending on where in the circuit the modulation occurred. As we have previously shown, OLM provides control over theta activation in the network due to its long time constants [18]. Reduced OLM 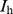 eliminated theta by removing this influence (Fig. 4, Fig. 5). This then allowed the PYR and BAS interactions to create strong, continuous gamma through ING and PING mechanisms. Increased OLM 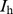 eliminated all activity by causing increased OLM activity which shut down activity in the other cells, OLM being an inhibitory cell type. Modulating 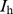 across all cell types had effects similar to those seen with OLM modulation, due to this strong governing influence of OLM.

Different neurotransmitters are likely to have differential effects on different cell types through effects on different receptors on the different cell types. Our modeling suggests likely cellular locations of neuromodulation targets for changing oscillation power and frequency. These could be tested by using immunohistochemistry to correlate the location of neurotransmitter receptor types with particular cell types. For example, it is known that noradrenaline is involved in 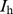 regulation [47]. In addition, recent experimental evidence demonstrates that acetylcholine modulates different features of 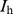 activity, including its sag amplitude [11], [12]. Interestingly, acetylcholine has also been shown to contribute to modulation of theta frequency over a range similar to that observed in our model [48].

The BAS cell is particularly involved in the genesis of gamma oscillations through the ING (BAS-BAS) and PING (PYR-BAS) mechanisms. Increased BAS cell 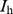 increased BAS activity and raised gamma power (Fig. 6, Fig. 7). This increase also slightly lowered gamma frequency, due to the increased duration of synaptic responses. The PYR cell is the only excitatory cell in the network and therefore plays a role in maintaining firing of all cell types. Increased PYR 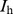 increased PYR 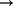 OLM activation and produced a monotonically increasing effect on both power and frequency of theta (Fig. 8, Fig. 9). Note that this apparent PYR 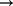 OLM effect was quite different than the more direct activation provided by increasing OLM 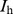. At the same time, the increased PYR 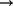 BAS activation produced a tendency to increased gamma power without consistent effect on frequency. The overall PYR effect was to tune both theta and gamma power together, distinct from other pharmacological effects where theta and gamma trade off [18].

Simultaneous modulation of PYR and BAS 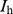 similarly comodulated power, while now shifting both frequencies consistently: gamma tuning towards lower frequency while theta tuned towards higher frequency with increased 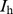 (Fig. 10, Fig. 11). Independent modulation of PYR and BAS 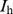 allowed flexible control of the frequencies and amplitudes of theta and gamma oscillations (Fig. 12a,b,c,d). We hypothesized that these modulations of theta and gamma oscillations could be utilized by functional mechanisms that are postulated to utilize linkages between theta and gamma to provide encodings such as phase precession in place cells [49], cross-frequency coupling (CFC) [35], [50]–[52], and gamma on theta phase for memory [20]. Indeed, our model demonstrated that shifting oscillatory modulations were effective in setting the CFC level, with increases evident at high theta PYR 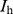 levels (Fig. 12e). We therefore predict the presence of distinct neurotransmitter receptor types in PYR and BAS cells which would allow 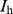 to be tuned independently, and therefore support flexible shifting of the CFC level.

Our model demonstrated that increased PYR 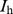 would increase PYR excitability, augment PYR 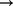 BAS feedforward activation via a PING mechanism, and thereby shift gamma activation to an earlier phase within the theta cycle. In the context of neural coding, the timing of pyramidal cell firing within a theta cycle has been hypothesized to allow the most relevant neurons for a particular stimulus to fire at earlier phases and then inhibit firing of other ensembles [53]. Our model suggests how modulation of 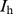 could enhance this contrast sensitivity by enhancing this initial activation. This is also consistent with recent experimental work that demonstrates the contribution of 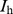 currents to hippocampal pyramidal neuron synchronization [54], which could cause downstream neurons to fire earlier, thereby modulating timing of gamma spikes.

Intracellular signalling can be used to modulate the degree to which 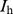 is regulated. This has been demonstrated experimentally in the heart [14], [55], and similar mechanisms may take place in neurons via neuromodulatory control [11]. This mechanism has been demonstrated in computer models of prefrontal cortex neurons [17]. In this process, the neuron is initially activated via feedforward excitatory inputs. With sufficiently strong activation, calcium is admitted. Subsequently, calcium binds to protein kinases (*e.g.,* cAMP) which bind to HCN and increase 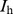 conductance, leading to increased excitability. Our model shows that in the neuronal network context, this process leads to frequency tuning, increased CFC, and earlier generation of gamma spikes by the activated cells. Due to long time constants of protein kinase binding with HCN, the effects of this initial activation could be used to prime a circuit's response to subsequent inputs.

Our current model remains limited by lack of explicit second messenger modeling and lack of detailed information about differences between HCN isoforms. In particular, cAMP, the second messenger which acts on 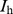, also has effects on K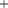 [56] or leak [6], [57] channels, which would also tend to change cell and network dynamics. Our HCN isoform modeling also remains limited, since we only included electrophysiological, and not second messenger, differences. Inclusion of second messenger signaling pathways will be of greatest value once further details are available concerning differences in second messenger responsitivity between the two major isoforms studied here. Further detail might also consider differences in phosphorylation states which provide further modulation of these channels [58].
