# Correction: *I_h_* Tunes Theta/Gamma Oscillations and Cross-Frequency Coupling In an *In Silico* CA3 Model

**DOI:** 10.1371/annotation/00ea7ef3-aafd-4675-a3cc-c5087816b4d6

**Published:** 2013-12-09

**Authors:** Samuel A. Neymotin, Markus M. Hilscher, Thiago C. Moulin, Yosef Skolnick, Maciej T. Lazarewicz, William W. Lytton

This article was republished on December 5th, 2013 because of missing mathematical symbols. Please download this article again to view the correct content. 

